# Alien Limb Phenomenon and Ideomotor Apraxia as Early Indicators of Sporadic Creutzfeldt‐Jakob Disease

**DOI:** 10.1002/mdc3.70070

**Published:** 2025-04-04

**Authors:** Gero Lueg, Ilka Kleffner, Markus A. Hobert

**Affiliations:** ^1^ Department of Geriatric Medicine, Marien Hospital Herne Ruhr University Bochum Herne Germany; ^2^ Department of Neurology University Hospital Knappschaftskrankenhaus, Ruhr University Bochum Bochum Germany; ^3^ Department of Neurology University Medical Center Schleswig‐Holstein, Campus Kiel and Christian‐Albrechts‐University of Kiel Kiel Germany; ^4^ Department of Neurology University Medical Center Schleswig‐Holstein, Campus Lübeck and University of Lübeck Lübeck Germany

**Keywords:** alien limb phenomenon, alien hand, apraxia, CJD, Creutzfeld‐Jakob

Creutzfeldt‐Jakob disease (CJD) is a rapidly progressive neurodegenerative disorder that encompasses symptoms such as dementia, myoclonus, visual or cerebellar impairment, pyramidal and extrapyramidal motor deficits, akinetic mutism and ataxia.^1^ The term “alien limb phenomenon” (ALP) describes involuntary motor activity in one limb while simultaneously experiencing a sense of estrangement from that same limb.^2^ ALP is a typical sign of corticobasal syndrome and has also been described in CJD.^3^ This clinical vignette presents the rapid clinical progression of a patient with left ALP and ideomotor apraxia as early symptoms of CJD.

Three weeks prior to admission, an independent, 83‐year‐old, right‐handed patient with no relevant medical history had a feeling of estrangement of his left hand, accompanied by a progressive loss of voluntary motor function. Within two weeks, he was unable to ride his bicycle and needed help with activities such as buttoning his shirt. Over time, he reported uncontrolled movements of his left hand that caused repeated dropping of objects. An outpatient magnetic resonance imaging (MRI) of the brain did not reveal any significant findings, specifically no stroke and focal brain atrophy.

On admission on December 20, 2021, the patient presented with dystonic posturing, occasional myoclonus and ataxia mainly affecting the left arm (Video 1, Segment 1). Other neurological signs like paresis, rigidity, tremor or aphasia were not present.

**Video 1:** Segment 1 shows dystonic posturing and myoclonus of the left hand and gait ataxia. Segment 2: “Paper‐Toss Test” for examination of unilateral ideomotor apraxia. Segment 3 shows loss of ambulation and rapid progression of dystonia and myoclonus.

Segment 2 of Video 1 shows a brief bedside test (“Paper‐Toss Test”) we use in our clinical routine to test for asymmetric apraxia. The patient is instructed to throw a piece of paper in a high arc with each hand separately. The patient shows mirror movements of both hands while attempting to grasp and release the paper. However, the movement is incomplete with the left hand. The left‐sided involuntary levitation and apraxia are consistent with a posterior variant of ALP, which commonly affects the non‐dominant side in CJD.^3^, ^4^ No typical early clinical signs of other ALP phenotypes were observed in this patient, suggesting the corpus callosum variant (manifestation of intermanual conflict behavior) or the frontal variant of ALP (involuntary grasping movements).^5^

Analysis of cerebrospinal fluid (CSF) revealed positive detection of protein 14–3‐3 without any other abnormalities such as lymphocytic pleocytosis. A new cerebral MRI showed typical cortical diffusion abnormalities, mainly involving the right parietal cortex, consistent with the ALP on the left side (Fig. 1). Electroencephalography (EEG) revealed episodic right hemispheric triphasic waves, and detection of pathologic prion protein (PrPCJD) using real‐time quaking‐induced conversion assay (RT‐QuIC) in the CSF confirmed the diagnosis.

**Figure 1:**
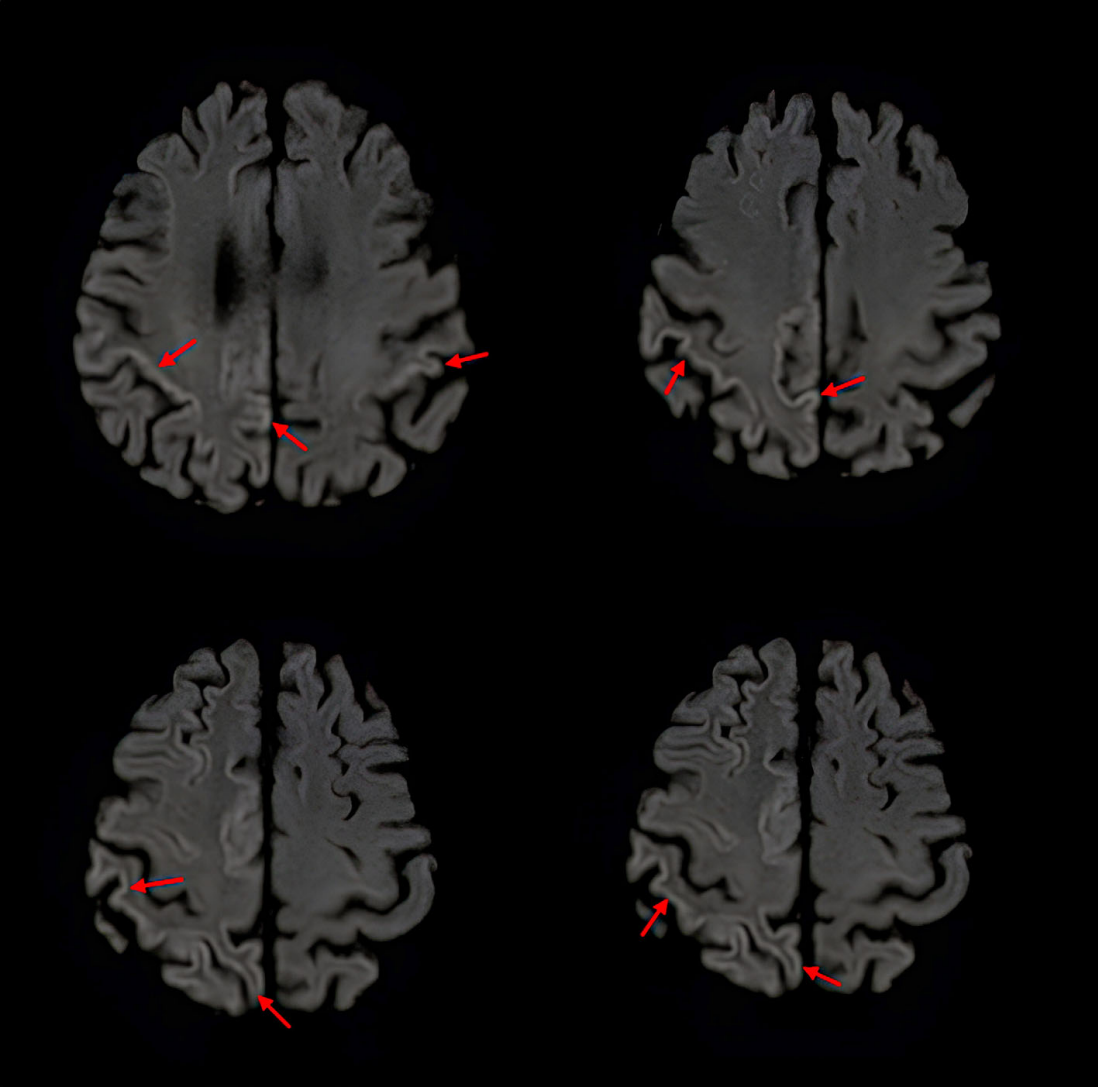
Diffusion‐weighted MRI (DWI) with right hemispheric and parietal accentuated diffusion restriction of the cortex (“cortical ribboning”). Specific anomalies are highlighted by red arrows.

On January 21, 2022, (Segment 3, Video 1), the patient's condition worsened, requiring use of a wheelchair due to severe gait ataxia and dystonia on the left side of the body. The myoclonus now spread to the right arm. The patient died four weeks later during an emergency readmission from the nursing home.

This case illustrates that the occurrence of ALP and unilateral ideomotor apraxia should be considered as early symptoms of sCJD and should be a reason for early diagnostic testing.

## Author Roles

(1) Research project: A. Conception, B. Organization, C. Execution; (2) Manuscript Preparation: A. Writing of the First Draft, B. Review and Critique.

G.L.: 1A, 1B, 1A, 1C, 2A

I.K.: 1B, 1C, 2B

M.A.H: 1C, 2B

## Disclosures

**Ethical Compliance Statement:** The Ethics Committee of Westphalia‐Lippe has reviewed the study (number 2024‐717‐f‐N) and confirmed that no further ethical approval is required in addition to the written informed consent provided by the patient. We confirm that we have read the Journal's position on issues involved in ethical publication and affirm that this work is consistent with those guidelines.

**Funding and Conflicts of Interest:** No specific funding was provided for this study. The authors declare that there are no relevant conflicts of interest.

**Financial Disclosures in the Past 12 Months:** The authors declare that no other financial disclosures are required.

## Data Availability

The data that support the findings of this study are available on request from the corresponding author. The data are not publicly available due to privacy or ethical restrictions.
